# Comparison between artificial intelligence-based and manual organ delineations in pretreatment computed tomography scans of prostate cancer patients: a visual grading study

**DOI:** 10.1093/rpd/ncaf184

**Published:** 2026-03-13

**Authors:** Eirini Polymeri, Åse A Johnsson, Olof Enqvist, Johannes Ulén, Jon Kindblom, Karin Braide, Hans-Jurgen Wiltz, Margareta Tanyasiová, Elin Trägårdh, Lars Edenbrandt, Henrik Kjölhede, Angelica Svalkvist

**Affiliations:** Department of Radiology, Institute of Clinical Sciences, Sahlgrenska Academy, University of Gothenburg, Medicinaregatan 3, Medicinareberget, 413 90, Gothenburg, Region Västra Götaland, Sweden; Department of Radiology, Sahlgrenska University Hospital, Blå Stråket 5, Region Västra Götaland, 413 45, Gothenburg, Sweden; Department of Radiology, Institute of Clinical Sciences, Sahlgrenska Academy, University of Gothenburg, Medicinaregatan 3, Medicinareberget, 413 90, Gothenburg, Region Västra Götaland, Sweden; Department of Radiology, Sahlgrenska University Hospital, Blå Stråket 5, Region Västra Götaland, 413 45, Gothenburg, Sweden; Department of Electrical Engineering, Chalmers University of Technology, Hörsalsvägen 9-11, Region Västra Götaland, 412 96, Gothenburg, Sweden; Eigenvision AB, Bredgatan 4, 211 30, Region Skåne, Malmö, Sweden; Eigenvision AB, Bredgatan 4, 211 30, Region Skåne, Malmö, Sweden; Department of Oncology, Sahlgrenska University Hospital, Blå Stråket 2, Region Västra Götaland, 413 45, Gothenburg, Sweden; Department of Oncology, Institute of Clinical Sciences, University of Gothenburg, Medicinaregatan 3, Medicinareberget, 413 90, Gothenburg, Region Västra Götaland, Sweden; Department of Oncology and Radiotherapy, Region Kronoberg, Central lasarett Växjö, Strandvägen 8, 351 85, Växjö, Sweden; Department of Oncology, Sahlgrenska University Hospital, Blå Stråket 2, Region Västra Götaland, 413 45, Gothenburg, Sweden; Clinical Physiology and Nuclear Medicine, Lund University and Skåne University Hospital, 221 85, Malmö, Region Skåne, Sweden; Department of Molecular and Clinical Medicine, Institute of Medicine, Sahlgrenska Academy, University of Gothenburg, Blå Stråket 5, 413 45, Region Västra Götaland, Gothenburg, Sweden; Department of Urology, Institute of Clinical Sciences, Sahlgrenska Academy, University of Gothenburg, Medicinaregatan 5, Medicinareberget, 413 90, Region Västra Götaland, Gothenburg, Sweden; Department of Urology, Sahlgrenska University Hospital, Blå Stråket 5, 413 45, Region Västra Götaland, Gothenburg, Sweden; Department of Medical Radiation Sciences, Institute of Clinical Sciences, Sahlgrenska Academy, University of Gothenburg, Medicinaregatan 5, Medicinareberget, 413 90, Region Västra Götaland, Gothenburg, Sweden; Department of Biomedical Engineering and Medical Physics, Sahlgrenska University Hospital, Blå Stråket 5, 413 45, Region Västra Götaland, Gothenburg, Sweden

## Abstract

This study aimed to evaluate the clinical acceptability of artificial intelligence (AI)-based organ segmentations on pretreatment CT images of prostate cancer patients using manual organ delineations as a reference. Paired AI-based segmentations and manual delineations of the prostate, urinary bladder, and rectum were evaluated by three observers, according to a 4-grade Likert-scale, based on quality criteria, developed through a Delphi process. Visual grading characteristics (VGC) analysis was performed. When comparing the ratings of AI-based (*n* = 360) and manual delineations (*n* = 360), the area under the VGC-curve (AUC_VGC_) was 0.36 (95% CI 0.27–0.44), 0.35 (95% CI 0.28–0.41), and 0.3 (95% CI 0.22–0.40) for the prostate, urinary bladder, and rectum, respectively, indicating inferior ratings for the algorithm. Few AI segmentations (8%) were considered clinically unacceptable, while in 67% no or minor changes were needed. Despite superior ratings for manual delineations, most AI-segmentations needed no or minor changes, indicating clinical acceptability.

## Introduction

Subjective image interpretation is one of the main sources of variability in the visual assessment of medical images [1, 2]*.* Organ delineation on images from computed tomography (CT) or magnetic resonance imaging (MRI) is a necessary procedure for radiation treatment planning of tumours. Several studies have shown that inaccurate organ delineation can lead to poorer clinical outcomes [3–5]. However, this task is highly observer-dependent and subject to inter- and intraobserver variability [6–8]. Inter- and intraobserver variability in organ delineation for radiation treatment planning are multifactorial in origin. Several possible solutions have been introduced, including standardized protocols, guidelines, and multimodal imaging [9]*.* However, inter- and intraobserver variability remain a challenge.

The development of artificial intelligence (AI) and its increasing use in oncological imaging and radiation oncology have had a dramatic impact in the past decade [10]. Several studies have shown the applicability of AI as a tool in organ delineations for radiation treatment planning of various forms of cancer, including prostate cancer (PCa) [11–15]. However, machine learning has been applied almost exclusively for prostate delineation on MRI for PCa diagnosis and staging, while its application to CT has been limited [16]*.* Moreover, the focus has been largely placed on the AI-based segmentation of target organs as well as organs at risk (OAR), which has reduced both manual delineation time and interobserver variability [12, 17]*.* Nevertheless, the quality and clinical acceptability of automated AI-based organ segmentation have not been adequately explored in PCa patients.

Most studies on AI-based organ segmentations have focused on geometric measurements, such as Hausdorff distance (HD), mean surface distance (MSD), and Dice–Sørensen Coefficient (DSC), showing good agreement between AI and the ground truth [18–24]. Yet, these metrics can lead to ambiguity regarding the clinical utility of the AI-based segmentations as they are strongly dependent on the ground truth. In addition, agreement between geometric measurements does not reflect clinical acceptability.

In 2007, Båth and Månsson [25] described the use of visual grading characteristics (VGC) analysis as a method to compare image quality using ordinal data. To date, this methodology has primarily been used for image quality assessment [26–29]. To the best of our knowledge, visual grading studies evaluating the quality and potential clinical use of AI-based organ segmentations for the radiotherapy planning of PCa patients are limited [22, 30–32].

Consequently, this study aimed to determine whether an AI algorithm can achieve clinically acceptable segmentations of the prostate and surrounding risk organs (OAR) on pretreatment CT scans using visual grading.

## Materials and methods

### Patient and image material

The study was approved by the Swedish Ethical Review Authority (2019-03205).

The CT scans of the patients included in the study were derived from a larger study cohort, which has been described in detail previously, along with the acquisition parameters used for the CT examinations [24]. The large dataset of CT scans comprised 1530 PCa patients at the Department of Oncology, Sahlgrenska University Hospital, Gothenburg, Sweden. Imaging was performed before radiation treatment planning between 2006 and 2018 [24].

**Figure 1:**
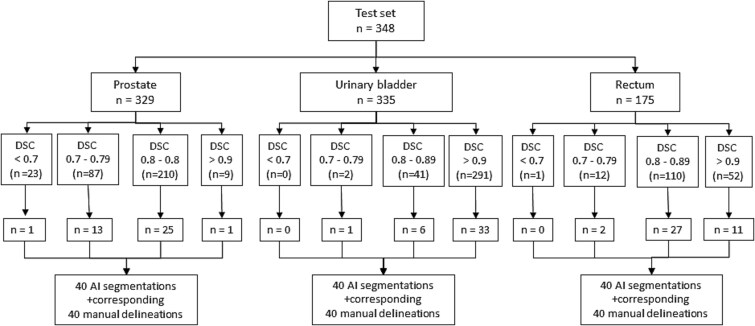
Flow chart of the collection of cases for visual grading, where the test set was a part of a previous study [24].

The AI algorithm used in the present study is a U-NET-based model and was developed by mathematicians for research purposes only. The method is not used in the clinical routine, and it was used for organ segmentation in CT images within a research framework described in detail in a previous study [24]. The algorithm was trained and validated on manual delineations of the image material described above, in order to create a test set of automated AI-based organ segmentations (prostate *n* = 329, urinary bladder *n* = 335, rectum *n* = 175) (Fig. 1) [24].

All manual delineations underwent quality control prior to inclusion in the study. The AI-based segmentations of the test set were compared with the corresponding manual delineations to calculate Dice Sørensen Coefficient (DSC) for each organ, which has been reported previously [24].

AI-based segmentations were divided into four categories according to DSC values: <0.7, 0.7–0.79, 0.8–0.89, and >0.9, describing the range of algorithm’s performance across organs. From this overall distribution, 120 organ segmentations (40 of each organ) were then randomly collected and included in the present study, to mimic the DSC distribution in the larger cohort, ensuring that both well-performing and lower-performing segmentations were represented. There were no rectum and urinary bladder segmentations with DSC < 0.7, while there were few prostate segmentations with DSC < 0.7 or > 0.9 (Fig. 1). For the visual grading, the assessments of the AI-based segmentations and their corresponding manual delineations were obtained for each organ (Fig. 1).

### Presentation of study data

The research platform “Recomia” (www.recomia.org) [33], a noncommercial and nonprofit program, was used for the visual evaluation of all organ segmentations. The AI algorithm segments the prostate and seminal vesicles with two separate labels and presenting these two labels to the observers would be a trivial way for them to determine if a delineation was AI-based and could lead to an unwanted bias. To ensure that manual and AI-based segmentations were presented in the same way to the observers for visual grading, all segmentations included the prostate gland and excluded the seminal vesicles, in accordance with the clinical delineation protocol used for the test dataset. If the manual prostate delineation for a case included the seminal vesicles, the AI-based segmentations of the prostate and seminal vesicles were merged into one label. If the manual prostate delineation for a case did not include the seminal vesicles, the AI-based segmentation of the seminal vesicles was not made visible to the observers. Consequently, the exclusion or the inclusion of seminal vesicles was consistent across AI and manual contours, minimizing potential bias in visual grading. The AI-based segmentations of the urinary bladder and rectum were presented without intervention.

### Visual assessment of AI-segmentation’s clinical acceptability

All information related to the organ segmentations was blinded. The AI segmentations and manual delineations were presented separately and in a randomized order to each observer. The observers rated the quality of each segmentation independently using a 4-grade Likert scale, as shown in Table 1. The rating scale was developed through a Delphi procedure, which is a well-established method for reaching consensus [34]. The group who contributed to this procedure consisted of eight specialists: two radiologists, one urologist, three radiation oncologists, one nuclear medicine physician, and one medical physicist. For the rectum, the criteria of acceptance included delineations up to the rectosigmoid junction. For the prostate, cases with and without delineations of the vesicles were included.

**Table 1:** Rating scale of clinical acceptance of organ segmentations for each organ.

Grading	Can the organ segmentation be clinically accepted/used for radiation treatment planning?
1.	Segmentation can be accepted/used for treatment planning
2.	No, would perform minor changes
3.	No, would perform major changes
4.	No, would delete and perform a new delineation

### Observers

Three radiation oncologists who work at different hospitals in the country participated in the study. Observer 1 (O1) is a specialist in urology and radiation oncology with 12 years of experience (K.B.), and Observer 2 (O2) and Observer 3 (O3) are radiation oncologists with 20 and 12 years of experience, respectively (M.T. and H.-J.W.). Detailed information on how to use the research platform was provided to the observers before the study. Three consensus meetings were also held prior to the study, to obtain a clear understanding of radiation treatment planning guidelines and interpretation of ratings, e.g. acceptance criteria regarding the delineation limits of the rectum. Before evaluating the segmentations included in the study cohort, the observers practised on a separate training material consisting of 10 cases per organ, analogous to those included in the actual study, and assessed according to the same procedure described above.

**Figure 2:**
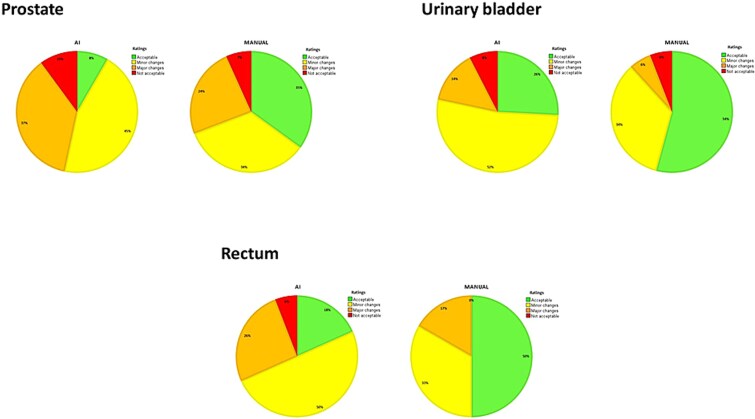
Pie charts showing the distribution of the visual grading of the observers for the 40 AI-based segmentations and their corresponding 40 manual delineations of each organ.

### Statistical analysis

Statistical analysis was performed using VGC analysis, a nonparametric, rank-invariant statistical method for analysing visual grading data [25]. In VGC analysis, paired ratings of organ delineation quality are compared by plotting the AI-based segmentation ratings against the manual delineation ratings to create a VGC curve. The separation between the ratings of manual and automatic segmentations is determined by the area under the VGC curve (AUC_VGC_), where an AUC_VCG_ of 0.5 represents no difference between the quality of the delineations, an AUC_VGC_ <0.5 represents higher ratings for the manual delineations, and an AUC_VGC_ > 0.5 represents higher ratings for the AI-based segmentations. The software VGC Analyzer [35] was used to determine AUC_VGC_ and statistically analyse the results. Using VGC Analyzer, the AUC_VGC_ can be determined using both the trapezoidal rule and binormal curve fitting. The statistical analysis can be performed for both paired and nonpaired data, and the results are presented for both the fixed reader situation (results applicable to the observers in the study) and the random reader situation (results applicable to a general population of observers).

To study the correlation between observer ratings for the AI-based segmentations of each organ and DSC, Spearman’s correlation coefficient was analysed using SPSS Statistics 29 (IBM). The level of significance was set to values outside the range of the 95% confidence interval (*P* ≤ .05).

## Results

In total, 120 AI-based organ segmentations and their corresponding manual delineations were evaluated by the observers. In the VGC analysis, the trapezoidal rule for curve fitting was used. Due to the small number of observers, the analysis was based on the fixed reader situation.

**Figure 3:**
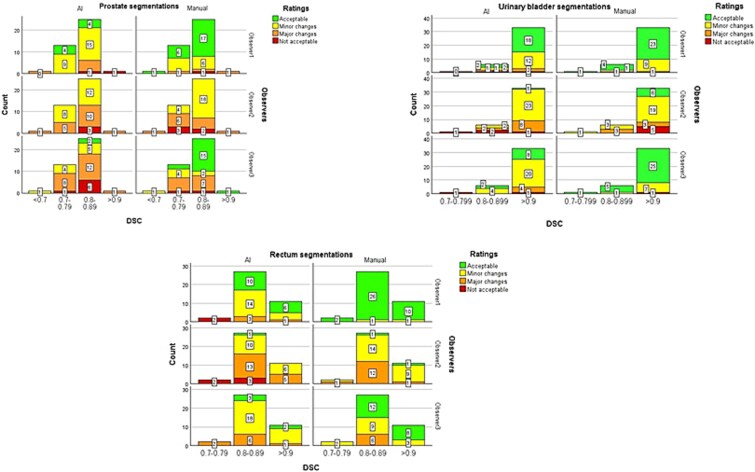
Distribution of the observer ratings in relation to DSC.

The ratings by the observers showed that a large proportion of all AI-based organ segmentations were acceptable for clinical use with no or minor modifications (67%), which corresponded to 53%, 78%, and 68% of the prostate, urinary bladder, and rectum segmentations, respectively. The corresponding results for the manual delineations were 69%, 88%, and 83%, respectively. Further, 37%, 14%, and 26% of the AI-based segmentations of the prostate, urinary bladder, and rectum, respectively, were assessed as acceptable with major changes, compared with 24%, 6%, and 17% of the manual delineations of the corresponding organs. A small number of delineations were evaluated as not acceptable, which corresponded to 10%, 8%, and 6% of the AI-based segmentations of the prostate, urinary bladder, and rectum, respectively, and 7%, 6%, and 0% of the manual delineations of the corresponding organs (Fig. 2). There was substantial variation in absolute ratings between the observers (Fig. 3). However, as VGC Analyzer [35] compare ratings individually for each observer, the variation between observers in absolute ratings will not influence the results of the statistical analysis.

**Figure 4:**
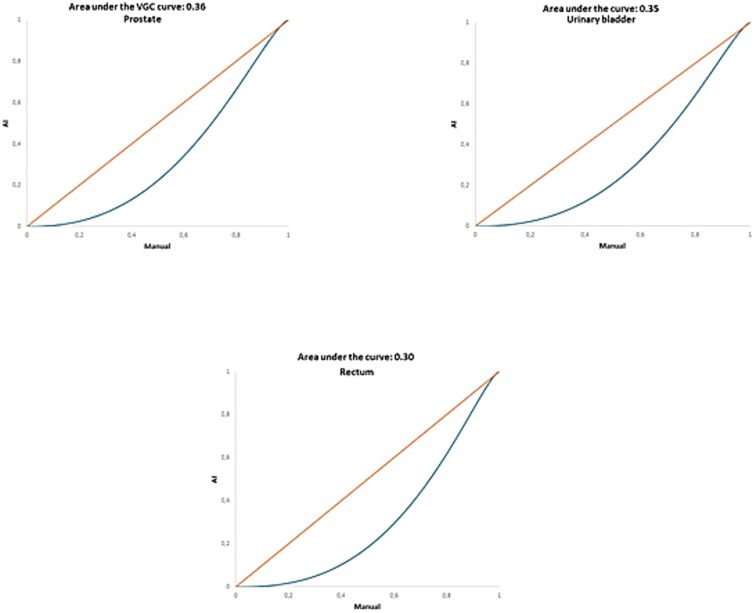
The area under the VGC curve (AUC_VGC_) for the prostate and the OARs.

For all AI-based organ segmentations, AUC_VGC_ values were significantly <0.5. The AUC_VGC_ for the prostate segmentations was 0.36 (95% CI 0.27–0.44), while the urinary bladder and rectum segmentations had AUC_VGC_ of 0.35 (95% CI 0.28–0.41) and 0.3 (95% CI 0.22–0.40), respectively (Table 2). The low AUC_VGC_ of the AI-based segmentations of all organs indicates that manual delineations were rated significantly better (Fig. 4).

**Table 2:** The area under the curve of the VGC analysis for each organ.

Organ of interest	AUC_VGC_^a^	95% CI^b^	*P*-value
Prostate	0.36	0.27–0.44	.002
Urinary bladder	0.35	0.28–0.41	< .001
Rectum	0.30	0.22–0.40	< .001

^a^Area under the VGC curve.

^b^95% Confidence interval.

**Figure 5:**
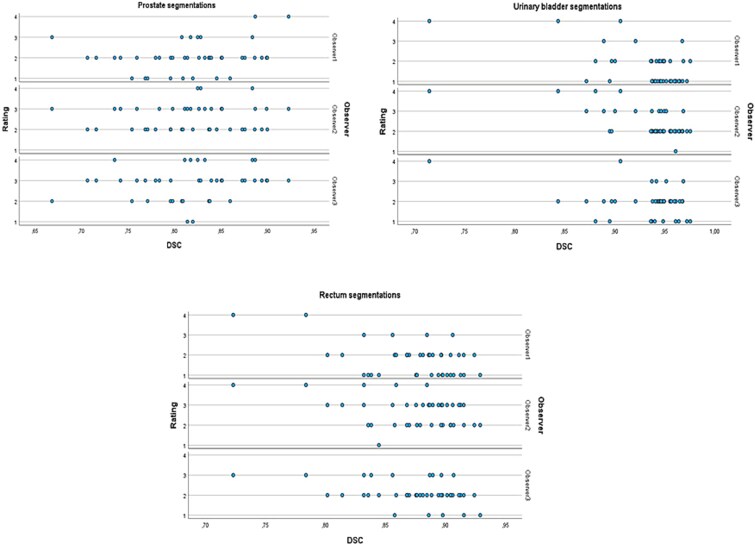
Scatter plots showing the correlation between the ratings by the observers and DSC.

Spearman’s coefficient analysis showed no correlation between the observer ratings and DSC for any organ AI segmentation (Fig. 5).

### Input from the observers

The observers provided feedback on the quality of segmentations and their ability to identify the AI-generated segmentations. Overall, observers noted certain cues that could potentially differentiate the segmentations, including adherence to anatomical organ boundaries, as AI-based segmentations occasionally contained segmentation errors that a human would not make. Other features of note were the asymmetry and angularity of the AI-generated segmentations, as well as the presence of extra contour lines into the neighbouring organs. Further, minor strikes were sometimes evident outside the organ of interest in the AI-based segmentations.

## Discussion

Visual grading by the observers showed that a large proportion of the AI-based segmentations was clinically acceptable with no or minor modifications, although manual delineations generally received higher ratings. The variability in absolute ratings for both manual delineations and AI-based segmentations, despite the consensus reached among observers on how to interpret the different rating alternatives [6–8, 36], highlights the inherent subjectivity and difficulty of pelvic organ delineation, rather than poor quality of the training data or inadequate reader assessment, that may exist in clinical reality.

An important consideration is the use of manual delineations as ground truth for the training of the AI algorithm, despite the absence of an absolute reference standard in pelvic organ delineation. Although all manual delineations underwent quality control before inclusion, they remain expert-dependent interpretations. While manual delineations remain the clinical standard, AI-based segmentations are still being perceived as inferior when deviating from conventional manual styles, despite inherently subjective expert evaluations [6–8]. Consequently, the observer’s ratings in this study reflected both the quality of the training delineations and the AI-generated segmentations.

Since the study material represented segmentations with different DSC, there was a large variation in the delineation qualities of the different organs. Yet, observer ratings for the AI segmentations showed no correlation with DSC, probably reflecting inter-observer variability. The visual grading by the observers suggested the need for some corrections of the AI-based segmentations, although the algorithm achieved accurate organ segmentations across various imaging datasets, despite the need for manual intervention. Low soft tissue contrast in CT contributes to poor differentiation between the bladder, the surrounding small intestine and the adjacent prostate [37]. Further, anatomical variations of the urinary bladder or the presence of nearby enlarged lymph nodes may cause difficulties in training the algorithm and lead to suboptimal AI-based segmentations, as reflected by the observers’ ratings.

Over the years, various upper limits for the delineation of the rectum have been applied in manual delineations, although the rectosigmoid junction has recently been established as the upper limit in international guidelines [38]. The 12-year span of the included examinations, during which radiotherapy guidelines were revised, together with anatomic and motility variations of the bowel contributed to lower ratings for the AI-based segmentations, of which only 18% were considered acceptable without any corrections, compared with 50% of the corresponding manual delineations. Nevertheless, the study material was deliberately chosen to include representative images from each DSC category and to reflect the overall heterogeneity within the source cohort of over 1500 patients [24].

Prostate ratings also varied greatly, even in images with high DSC values, mainly because of varying manual delineations used as training material. Delineation is problematic at the apex of the prostate and near the seminal vesicles as it is difficult to differentiate between soft tissues in this area [7, 39, 40]. In a recent study, this issue affected the delineations of the prostate, resulting in differing AI- and manual segmentations [23]. Gardner *et al*. have also encountered this issue when comparing human consensus organ delineations and Deformable Image Registration-generated (DIR) delineations [41]. The results of this study showed variation between the delineations, suggesting that manual correction is necessary for their clinical use.

Several recent studies have focused on the automated segmentation of target volumes and PCa tumours, including the seminal vesicles and the prostatic bed [42–44]. Still, studies evaluating the clinical usefulness of AI-based organ segmentations are limited. A recent study demonstrated successful AI-based segmentation of both the target organ and OAR in images of PCa patients, the results of which showed good agreement with the corresponding manual delineations on pretreatment CT scans [24]. The present study further explored the visual grading of AI-based organ segmentations in PCa patients with respect to their future clinical utility and—despite successful AI-based segmentations—highlighted the high variability between visual assessments. Schreier *et al*. [45] have also evaluated the segmentation quality of a deep-learning tool that was applied to the prostate and OAR before radiation treatment and showed comparable DSC values to those of the aforementioned study [24]. Still, Schreier *et al*. included fewer patients and mostly cone-beam CT (CBCT) images, which provides lower tissue contrast compared with CT [46]. This may have contributed to poorer recognition of the algorithm’s segmentation errors compared to CT. Radici *et al*. have also reached the same conclusion regarding CT and CBCT segmentations of the prostate, urinary bladder, and rectum (10 per organ) and also showed no significant differences in geometric measurements between AI-based segmentations and consensus manual organ delineations [23]. The present study included 40 AI-based CT segmentations of each organ and visual grading was performed.

In another study, Urago *et al*. [47] compared AI- and atlas-based organ segmentations of CT images and, after estimating them visually, showed that the AI-derived segmentations needed fewer manual corrections. In the present study, the ground truth consisted of manually delineated clinical CT scans, some of which were also combined with MRI, making the delineations more accurate.

A recent study by Duan *et al*. [22] also investigated the clinical acceptability of AI-segmentations based on visual assessments. However, the algorithm used in the present study was trained on a larger dataset, and DSC values were also taken into account for the analysis, which allowed for the comparison between geometric measurements and visual assessments. Moreover, observers from different clinics with varying experience levels participated in the present study, which reflects the existing variability in clinical practice.

A challenge regarding the use of AI is observer vigilance and the risk of potential identification of AI as the source of organ segmentation. In the present study, subtle segmentation errors made by the algorithm were noted by the observers, particularly at the rectosigmoid junction or near organ boundaries. This could potentially lead to the association of these cues with AI-based segmentations, leading to unwanted bias. Ivarsson and Lindwall [48] described the effect of human attitude towards AI, highlighting a tendency of individuals to align their personal beliefs about an AI product’s origin and accuracy with their perceptions. However, this issue was outside the scope of the present study. A structured study methodology is needed to evaluate whether observers can identify AI-generated segmentations in this clinical context.

The current study had some limitations. The cohort consisted of a heterogeneous set of CT images obtained between 2006 and 2018, during which both image quality and the delineation guidelines varied. Moreover, MRI was not yet routinely used in all patients, although multimodal imaging improves pelvic organ visualization and segmentation accuracy as well as irradiation doses [38, 49–52]. However, variations in the manual delineation of prostate tumours have also been observed in MRI [36, 53, 54]*.*

Although a limited number of images was included from the initial cohort, representative cases of all DSC values were included for visual grading. Only 40 AI-based segmentations from each organ were presented to the observers, yet, this number exceeded the size of the patient cohort (*n* = 10) in the study by Radici *et al*. [23]. However, future studies with more cases and observers would further evaluate the clinical usefulness of AI-based segmentations.

In the present study, the different organs were presented to the observers separately rather than simultaneously as in clinical practice. This allowed for an independent assessment of each organ without possible bias from the neighbouring structures.

Although the Likert scale used in this study primarily functioned as a binary measure of clinical acceptability, this approach was chosen to reflect a clinically relevant decision-making process. Additionally, the fact that the obtained ratings could be treated as ordinal data enabled the use of VGC analysis as a complementary evaluation method.

## Conclusion

In conclusion, the majority of AI-generated organ segmentations was subjectively rated as clinically acceptable for treatment planning of PCa patients, where no or minor modifications were considered necessary. Only a small number of the AI-based segmentations were not considered clinically acceptable. The comparative visual analysis underscored the potential of combining AI technology and human expertise, yet, highlighted the issue of subjective variability between observers regardless of segmentation method used. For clinical implementation, future studies could define objective criteria for acceptable AI segmentations and specify the level of manual adjustment while still retaining clinical benefit of an AI-assisted workflow.

## Abbreviations

CT, computed tomography; MRI, magnetic resonance imaging; OAR, organs at risk; AI, Artificial Intelligence; PCa, prostate cancer; HD, Hausdorff distance; MSD, Mean surface distance; DSC, Sørensen–Dice similarity coefficient; VGC, visual grading characteristics; AUC_VGC_, Area under the VGC curve.
